# Approach of automatic 3D geological mapping: the case of the Kovdor phoscorite-carbonatite complex, NW Russia

**DOI:** 10.1038/s41598-017-06972-9

**Published:** 2017-07-31

**Authors:** A. O. Kalashnikov, G. Yu Ivanyuk, J. A. Mikhailova, V. A. Sokharev

**Affiliations:** 1 Geological Institute of Kola Science Centre, Russian Academy of Sciences (GI KSC RAS), 14 Fersman Street, Apatity, Murmansk Region 184209 Russia; 2 Kola Science Centre of Russian Academy of Sciences (KSC RAS), 14 Fersman Street, Apatity, Murmansk Region 184209 Russia; 3 JSC “Kovdorskiy GOK”, 5 Sukhachova Street, Kovdor, Murmansk Region 184141 Russia

## Abstract

We have developed an approach for automatic 3D geological mapping based on conversion of chemical composition of rocks to mineral composition by logical computation. It allows to calculate mineral composition based on bulk rock chemistry, interpolate the mineral composition in the same way as chemical composition, and, finally, build a 3D geological model. The approach was developed for the Kovdor phoscorite-carbonatite complex containing the Kovdor baddeleyite-apatite-magnetite deposit. We used 4 bulk rock chemistry analyses – Fe_magn_, P_2_O_5_, CO_2_ and SiO_2_. We used four techniques for prediction of rock types – calculation of normative mineral compositions (norms), multiple regression, artificial neural network and developed by logical evaluation. The two latter became the best. As a result, we distinguished 14 types of phoscorites (forsterite-apatite-magnetite-carbonate rock), carbonatite and host rocks. The results show good convergence with our petrographical studies of the deposit, and recent manually built maps. The proposed approach can be used as a tool of a deposit genesis reconstruction and preliminary geometallurgical modelling.

## Introduction

Methodology of spatial analysis has been developed significantly in recent years^1–4^. However, geological maps highly depend on subjective factors such as theoretical basis of authors, accepted concept of geological structure genesis, etc.

Most of three-dimensional geological modelling methods are based on cross-sections built manually using borehole data, and/or serious expert solutions accepted during the modelling process, e.g. refs 5 and 6. The method based on potential-field interpolation and geological rules^7^ seems to be one of the best methods of 3D geological modelling that is less affected by subjective factors. However, development of *a priori* geological rules (age relations and rock sequences) often presents some difficulty, especially in case of complicated deposits such as complex magmatic, metasomatic, hydrothermal, etc. Such deposits usually have continuous transitions and uncertain age relations between different rock types, and questionable genesis. Another nearly data-driven method for geological modelling is recently presented by Hajsadeghi and colleagues^8^.

**Figure 1:**
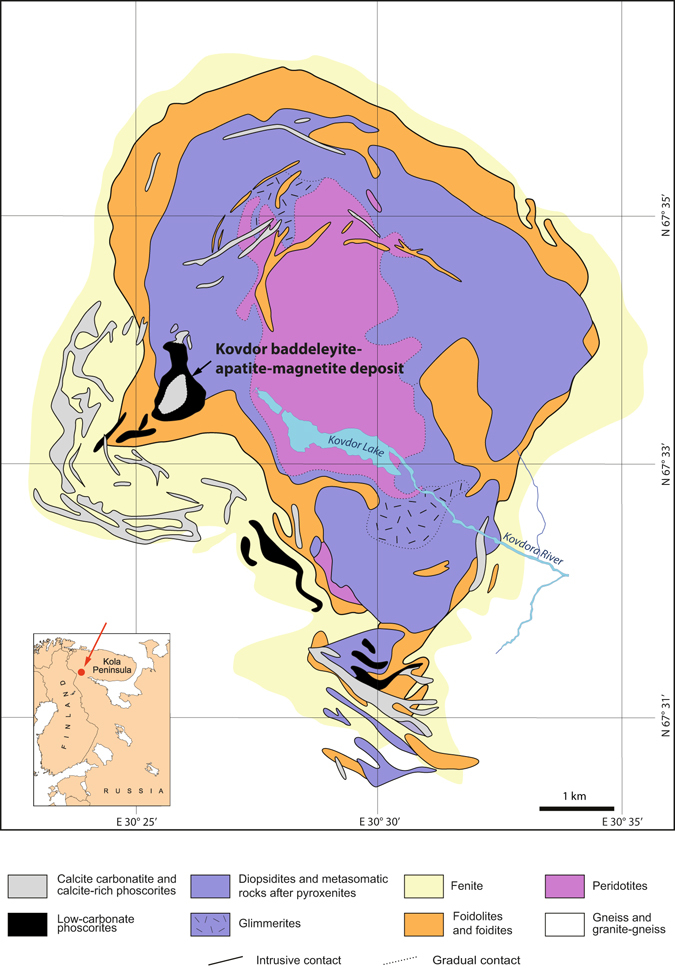
Geological map of the Kovdor massif (modified from ref. 15).

A new method of 3D geological mapping has appeared as a result of our attempts to minimize the influence of the controversial aspects on geologic modelling of the Kovdor phoscorite-carbonatite complex hosting the Kovdor baddeleyite-apatite-magnetite deposit (Murmansk region, NW Russia, Fig. 1).

Besides, there is a problem: ore classification for geological and geometallurgical mapping must be based on the rock modal composition; but mineralogical studies of numerous samples are too expensive and time-consuming (therefore, only chemical composition of ores has been analyzed using a dense network of sampling points). This problem is important for mineral engineering, processing mineralogy and geometallurgy, because mineral processing is based on the properties of ore minerals. It is usually solved by indirect mineralogical characterization and element-to-mineral conversion, which are the cheapest and fastest methods^9–12^. The method of indirect mineralogical characterization enabled us to distinguish 16 ore types using contents of the main minerals (forsterite, apatite, magnetite and calcite) calculated from bulk rock chemistry analyses of Fe_magn_, P_2_O_5_, CO_2_ and SiO_2_. Thus, the proposed approach can be used as a tool of preliminary geometallurgical 3D-modelling.

## Geological setting

The Kovdor apatite-magnetite-baddeleyite deposit is situated in SW part of the Kovdor alkaline-ultrabasic massif with carbonatites. The deposit is a concentric-zonal pipe of barren (outer zone) and ore-bearing phoscorites (axled zone) filled with veins of calcite and dolomite carbonatites. Ore-bearing phoscorites are represented by continuous series of rocks consisting of apatite, magnetite, calcite and forsterite. Please see our previous publications for details^13–15^.

There are two points of view on the genesis of the Kovdor deposit: plutonic and hydrothermal-metasomatic. Plutonic hypothesis interprets different phoscorites and ore types as different intrusive phases; the main process of ore genesis was crystallization from magmatic melt^16, 17^. According to the hydrothermal-metasomatic hypothesis, phoscorites are the product of hydrothermal-metasomatic alteration of initially intrusive body and crystallization from postmagmatic solutions and emanations; the main process of ore genesis was a replacement^18–20^. Discussion of the Kovdor phoscorite and carbonatite genesis is not finished^13, 14, 21^; therefore, the geological modelling method with minimum *a priori* genetic notions is especially required for the Kovdor deposit.

## Results

### A general methodology of the 3D geological mapping

The method of automatic 3D geological mapping involves the following actions:

1. Collect representative samples from a deposit. Specify chemical composition of rock samples and percentage of rock-forming minerals.
2. Develop an appropriate petrographic classification of rocks based on major minerals modal composition, corresponding to generally accepted classifications (e.g. IUGC classification^22^ for igneous rocks) and/or geometallurgical requirements. Define a rock type for each sample of “learning set” in accordance with the classification.
3. Define relationships between chemical and mineral composition of rocks, in one way or another. We propose mineral norm (normative mineral contents) calculation, statistical methods (artificial neural network, multiple regression, etc.) and logical computation of rock type boundaries (see the next section). Derive an algorithm defining a rock type by its chemical composition in accordance with the relationships specified above.
4. Build a set of block models showing a spatial distribution of the elements concentrations and merge these into one table. All essential conditions for geostatistical analysis and interpolation shall be maintained. General faults are taken from the historical database or revealed from interpolation patterns. Secondary (intraformation) faults are ignored.
5. Evaluate a rock type for each block in accordance with the algorithm specified above, and build a 3D block model with the evaluated rock type. It will be a final result.
6. Testing the results.

### Implication for the Kovdor phoscorite-carbonatite complex

Results will be presented in accordance with the actions listed in the section *A general methodology of the 3D geological mapping*.

1. Sampling of the deposit rocks and ores are described in the section *Materials and Methods*. Since a sample size for chemical analysis is three orders bigger than that for petrographic analyses (16 m and 3 cm, correspondingly), we selected petrographic samples (thin sections) representatively reflecting the corresponding core interval. To analyze the relationship between mineral and chemical compositions, we excluded petrographic samples taken from the ends of core intervals, and from inhomogeneous intervals. So, we excluded 276 petrographic samples, and finally analyzed 274 samples. Besides, these excluded samples were used for testing further results of rock type ‘prediction’ by their chemical composition. Statistical parameters of components used for automatic 3D geological mapping (SiO_2_, Fe_magn_, P_2_O_5_, CO_2_) are shown in Table 1.

**Table 1:** Statistical parameters of components used for automatic 3D geological mapping, wt. %.

Rock (number of samples)		SiO_2_						Fe_magnetic_						P_2_O_5_						CO_2_					
		Mean	Median	SD	Cf. var.	Min	Max	Mean	Median	SD	Cf. var.	Min	Max	Mean	Median	SD	Cf. var.	Min	Max	Mean	Median	SD	Cf. var.	Min	Max
Phoscorites	F (8)	31.1	31.6	4.2	14	24.5	36.0	8.4	8.4	3.2	38	3.5	14.1	3.0	3.3	0.9	31	1.2	4.0	4.3	3.3	2.9	68	1.4	9.0
	AF (30)	25.4	24.8	4.1	16	17.6	34.4	9.9	10.6	3.1	31	4.4	14.8	7.0	6.8	1.7	24	4.1	11.3	3.5	3.1	1.8	52	0.8	8.7
	M (4)	6.1	6.2	0.7	11	5.2	6.6	38.9	39.9	8.5	22	29.1	46.5	3.5	3.6	0.5	16	2.7	4.0	6.9	5.7	5.6	82	2.3	13.9
	MF (13)	14.7	14.1	6.7	46	7.3	27.1	29.3	29.0	7.9	27	16.0	41.1	1.8	1.8	1.0	56	0.2	3.4	5.5	4.6	3.6	66	1.3	13.0
	MAF (69)	13.7	13.0	5.6	41	7.0	26.2	24.7	26.0	7.1	29	12.4	39.2	8.8	8.8	2.9	33	4.0	15.8	2.8	2.4	1.5	53	0.7	7.8
	MA (19)	7.0	6.1	4.2	60	3.4	22.1	31.2	32.8	5.6	18	16.0	42.0	9.4	10.1	2.3	25	5.4	13.9	4.5	4.6	2.3	52	1.2	9.3
	CMAF (9)	8.4	8.5	1.0	12	7.2	10.0	23.8	22.0	5.9	25	14.8	34.2	6.8	7.2	2.0	29	2.2	9.1	11.2	12.0	2.6	23	7.3	14.9
	CMF (3)	7.7	7.8	0.5	7	7.0	8.1	30.4	26.8	9.0	30	23.7	40.6	1.1	1.2	0.6	53	0.4	1.5	13.4	14.1	3.6	27	9.5	16.7
	CAF (3)	11.3	10.3	1.8	16	10.2	13.4	12.6	12.3	0.9	7	11.8	13.5	8.3	8.7	1.1	13	7.1	9.1	13.9	14.1	0.7	5	13.2	14.4
	CM (9)	5.6	5.9	0.7	13	4.4	6.3	24.2	25.9	3.7	15	18.4	28.6	2.3	2.6	1.0	42	1.2	3.8	18.1	17.5	1.8	10	16.1	20.9
	CA (9)	3.5	3.7	0.8	22	2.3	4.5	10.7	11.2	2.0	19	7.1	13.7	8.0	8.1	2.0	25	4.4	10.5	23.9	22.1	4.5	19	18.2	31.7
	CMA (22)	5.0	5.1	0.9	19	3.2	6.7	23.7	24.1	4.6	19	15.1	32.1	7.1	7.6	2.0	28	1.7	10.2	14.0	13.7	3.8	27	8.2	24.1
Carbonatite (42)		2.7	2.2	1.8	66	1.0	11.3	8.3	7.8	5.3	64	1.2	19.2	5.0	4.4	1.8	37	1.2	9.7	28.9	29.3	5.4	19	19.3	38.4
Host rocks (38)		38.3	38.7	4.1	11	30.1	50.1	3.3	2.5	1.7	53	1.3	8.1	1.2	1.1	0.4	37	0.3	1.9	5.2	5.0	1.7	33	1.9	8.9

SD is standard deviation. Cf. var. is coefficient of variation.

Letters C, M, A, F are mineral indexes in phoscorite name: calcite, magnetite, apatite and forsterite, correspondingly (classification is shown in Fig. 2).
2. The petrographic classification of the Kovdor’s phoscorites based on mineral composition was developed in our earlier paper^14^, and shown in Fig. 2. The classification tetrahedron includes both carbonatites (carbonate minerals content is above 50%) and phoscorites. Phoscorites were divided into varieties according to the content of magnetite, apatite and forsterite. So, a name of each rock type is a combination of four mineral indexes: C (carbonates, mainly calcite), M (magnetite), A (apatite) and F (forsterite); the order of indexes does not reflect the mineral percentage. Hence, there are 15 rock types of carbonatite–phoscorite series: forsteritite (F), AF (forsterite-rich phoscorites); apatitite (A), magnetitite (M), MA, MF, AF, MAF (low-calcite magnetite-rich phoscorites); CA, CM, CF, CAF, CMA, CMF, CMAF (calcite-rich phoscorites), carbonatite.

**Figure 2:**
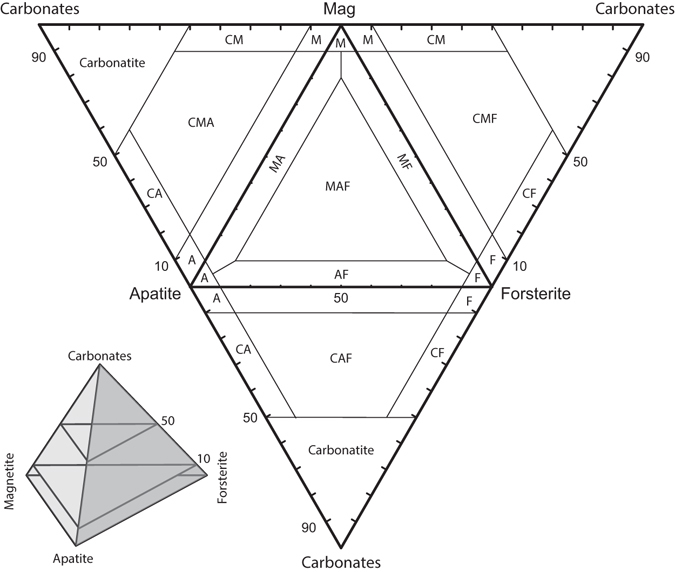
Classification scheme of the phoscorite-carbonatite series rocks. The classification tetrahedron (left bottom) and its net. Indexes in phoscorite names: A – apatite, F – forsterite, M – magnetite, and C – carbonates (calcite and dolomite) (modified ref. 14).
3. We used 4 techniques for chemistry-to-mineral conversion: mineral norms calculation, multiple regression; artificial neural network; and logical computation. We have performed centered log-ratio transformation of the bulk-rock chemistry data before multiple regression and artificial neural network have been applied.

#### Normative mineral content (norms) calculation

The rock-forming minerals of the Kovdor’s phoscorites and carbonatites (i.e. carbonates, mainly calcite, magnetite, apatite and forsterite) have distinct chemical composition. Ideal composition of calcite is CaO = 56.03%, CO_2_ = 43.97%; hydroxilapatite is CaO = 55.82%, P_2_O_5_ = 42.39%, H_2_O = 1.79%; magnetite is Fe = 72.4%; and forsterite is MgO = 57.29%, SiO_2_ = 42.71%. So, it seems that bulk-rock chemistry conversion based on ideal composition of the minerals would lead to good representation of mineral composition. However, it is right for calcite, magnetite and apatite, but not for forsterite. Firstly, because there are other silicates in the deposit, predominantly phlogopite – on average, from 1 to 5 vol. % and rarely up to 10 vol. %. Secondly, delineation of host rocks is the aim of the automatic mapping, too; and host rocks mainly consists of silicates (nepheline, aegirine-augite, diopside, richterite, K-feldspar, forsterite, phlogopite, etc.). As norm calculations cannot divide forsterite-rich phoscorite (forsteritite, AF, MF etc.) and host rocks, we have cut off a volume of norm calculation by conventional border of P_2_O_5_ and Fe_total_.

Algorithm of mineral norm calculation is following. First, we calculate content of calcite (C), magnetite (M), apatite (A) and forsterite (F) based on their ideal composition:

$$\begin{array}{rcl}{\rm{C}} & = & ({{\rm{CO}}}_{2}^{\mathrm{bulk}-\mathrm{rock}}\ast 100)/43.97;\\ {\rm{M}} & = & ({{\rm{Fe}}}_{{\rm{magn}}}^{\mathrm{bulk}-\mathrm{rock}}\ast 100)/72.40;\\ {\rm{A}} & = & ({{\rm{P}}}_{2}{{\rm{O}}}_{5}^{\mathrm{bulk}-\mathrm{rock}}\ast 100)/42.39;\\ {\rm{F}} & = & 100-({\rm{C}}+{\rm{M}}+{\rm{A}});\end{array}$$

and then determine rock type in accordance with the classification by mineral composition (Fig. 2).

#### Multiple regression

We performed multiple regression prediction (a general linear model) of calcite, magnetite, apatite and forsterite percentage by the bulk rock chemistry with centered log-ratio transformation of the bulk chemistry to avoid spurious correlation in closure numerical systems^23, 24^. The method of multiple regression is forward stepwise, intercept is included in the model; tolerance is 0.0001. Coefficients of unknown parameters (β) and linear multiple correlation R of the regression are shown in Table 2. Only calcite prediction showed a reasonable coefficient of multiple correlation, and prediction of the other minerals was rather bad.

**Table 2:** Multiple regression for prediction of the mineral contents by bulk-rock chemistry of the Kovdor phoscorite-carbonatite complex.

	β					Multiple R
	Intercept	SiO_2_	Fe_magn_	P_2_O_5_	CO_2_	
Calcite	28.81	—	—	19.87	6.14	0.725
Magnetite	14.58	1.65	16.59	−3.01	—	0.477
Apatite	27.58	—	−2.34	9.73	−4.75	0.483
Forsterite	23.25	13.77	—	2.33	—	0.536

#### Artificial neural network

We have built some neural networks of different architectures (multilayer perceptron and radial basis function network) for revelation of relationship of bulk rock chemistry and rock type, i.e. it has been a classification problem, not a regression problem as in paragraph 3.2. It has been a supervised learning: train set included 70% of samples, testing set 15% and validation set 15%. The best model is radial basis function network with 4 input neurons, 26 hidden neurons, 14 output neurons, with Gaussian hidden activation and Identity function for output activation, error function is the sum of squares. A training performance of the network is 74.05%, test performance is 71.79% and validation performance is 74.36%. The trained neural network is included in Supplementary Materials 1.

#### Logical computation of rock type boundaries

**Figure 3:**
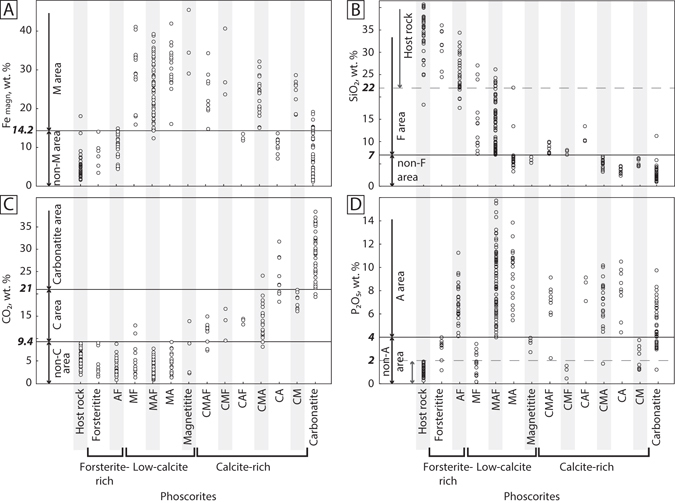
Boundaries between phoscorite types, carbonatite and host rocks in terms of bulk-rock chemistry. X-axis is rock name (letters C, M, A, F are mineral indexes in phoscorite name: calcite, magnetite, apatite and forsterite, correspondingly, classification is shown in Fig. 2). Y-axis is chemical composition of rocks; bold numbers are values for logical evaluation of a rock type (Fig. 4).

Comparison of chemical composition of different rock types demonstrated rather clear distinction. The boundary conditions specifying the use of mineral indexes (C, M, A, F) in rock names are determined as follows (Fig. 3): (a) plot diagrams: rock types (i.e. rocks classified by their major mineralogy) versus content of chemical elements in the rock; (b) specify the limits of each element content corresponding to the classification limits of mineral content.

First, carbonatites and host rocks were distinguished. Rocks with CO_2_ ≥ 21 mass % (Fig. 3C) were associated with carbonatites. The host rocks of the Kovdor deposit consist mainly of essential silicate (diopsidite, foidolite, apogneiss fenite, phlogopitite, skarn-like vesuvianite-garnet rocks, etc.). They are well separated from low-forsterite phoscorites by SiO_2_ content (separation line is SiO_2_ = 22 mass %, Fig. 3B); but the host rock is overlapping forsterite-rich phoscorites in SiO_2_ plot. So, to separate the host rock, we introduced the second discrimination parameter: P_2_O_5_ < 2 mass % (Fig. 3D). All rocks associated with host rocks in terms of their real mineral content were also characterized as host rocks in terms of their SiO_2_ and P_2_O_5_ content.

One can see that calcite-bearing phoscorites (with index “C”) are well separated from low-calcite ones (without index “C”) by separation line at CO_2_ = 9.4 mass %. Only three samples without index “C” (MF and Magnetitite) lie above the line. Phoscorites with index “M” (magnetite ores) are well separated by the line at Fe_magn_ = 14.2 mass % – only one magnetite-bearing sample (MAF) lies in “non-M” area, and one magnetite-free sample (AF) lies in “M area” (Fig. 3A). Two apatite-bearing outliers (CMAF and CMA) essentially below the line separating “A area” and “non-A area” (P_2_O_5_ = 4 mass %), and two forsterite-free outliers (MA) in “non-F area” (separation line is SiO_2_ = 7 mass %) are probably caused by unrepresentative interval characterization by thin section; though we excluded 50% of initial sample set.

**Figure 4:**
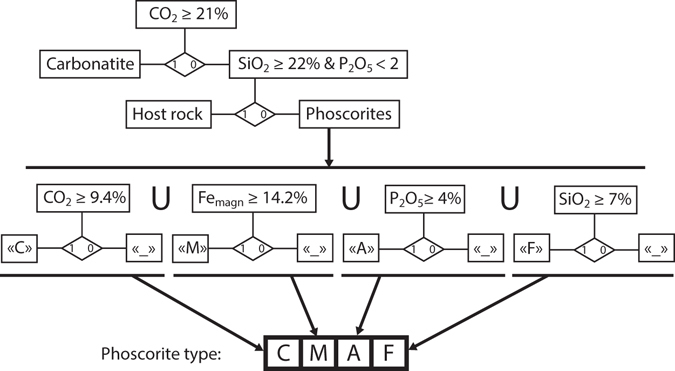
Flowchart of logical evaluation of a rock type.

Separation of mineral indexes by chemical composition of rocks allowed us to create a logical scheme for automatic determination of a rock type. Logical evaluation sequence for rock type determination by chemical composition is shown on the following flowchart (Fig. 4).

Beside 16 rock types (14 types of phoscorites, carbonatite and host rock), “unrecognized” type was delineated. “Unrecognized” value appears if the set of mineral indexes becomes “_ _ _ _” or “C _ _ _”. We assume the blocks with this value probably represent either dolomite-rich rocks (dolomite carbonatite or magnetite-serpentine-dolomite ore) or artifact. Anyway, unrecognized blocks are less than 0.1% of final geological 3D block model.

The set of logical formulas in MS Excel format is included in Supplementary Materials 2.

**Figure 5:**
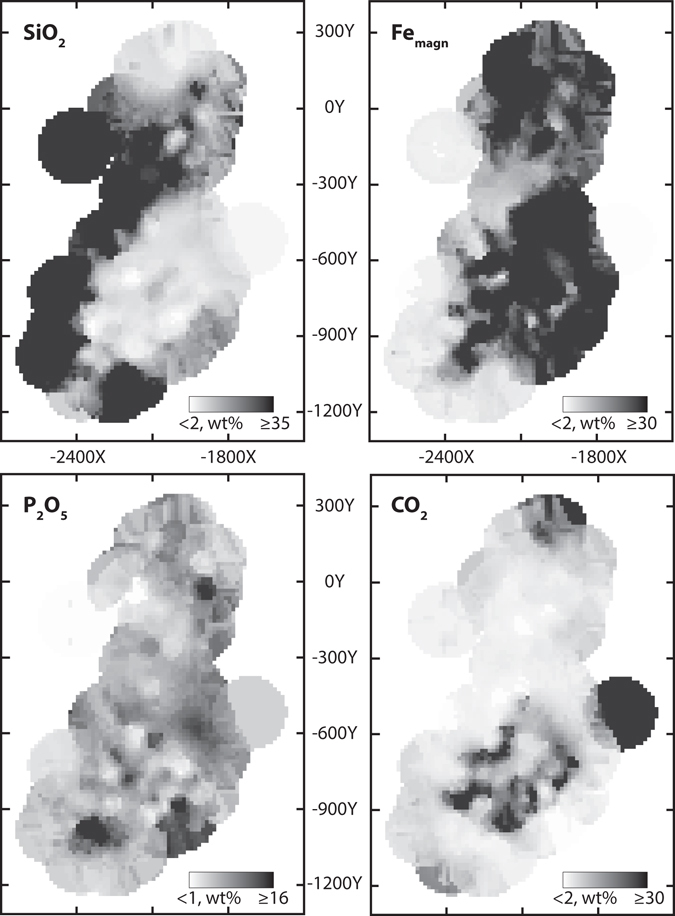
Spatial distribution of SiO_2_, Fe_magn_, P_2_O_5_, CO_2_ concentrations in rocks. Horizontal section, level −100 m. The maps were generated by Micromine 2016, commercial license (http://www.micromine.com).

3D block models of spatial distribution of SiO_2_, Fe_magn_, P_2_O_5_, CO_2_ were built. The parameters were interpolated by ordinary kriging. As a result, we generated 4 block models with 249437 blocks each. Their horizontal (−110 m) sections are shown in Fig. 5.

**Figure 6:**
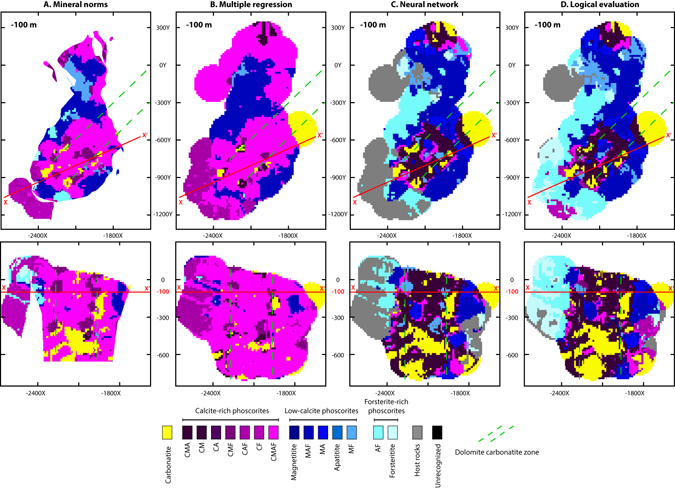
3D geological models of the Kovdor deposit automatically built by different methods from drillcore bulk-rock chemistry: (**A**) – by calculations of mineral norms; (**B**) – by multiple regression; (**C**) – by artificial neural network; (**D**) – by logical evaluation. Horizontal section −100 m (top) and cross-section X–X’. Red lines – location of sections. Green dashed line is the zone of dolomite carbonatite veins (Fig. 7). The 3D models were generated by Micromine 2016, commercial license (http://www.micromine.com).

The block models were integrated in a single file, and then each of developed predicting models (i.e. mineral norms, multiple regression, neural network and logical computation) was applied to each block. As a result, we built four 3D models of spatial distribution of the rock types, i.e. geological model. Horizontal and transverse vertical sections of the block models are shown in Fig. 6.

**Figure 7:**
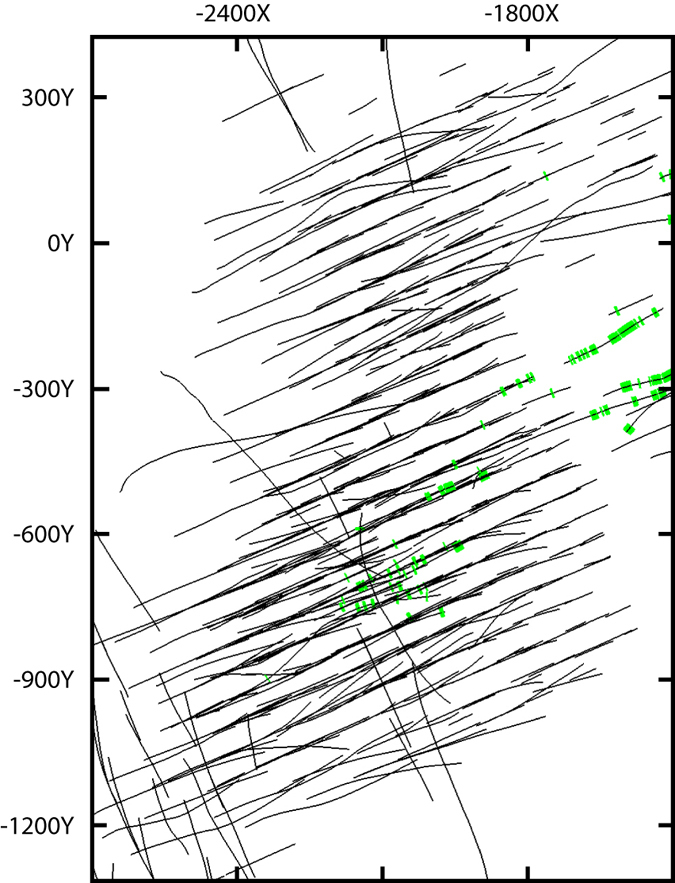
Borehole intervals with dolomite carbonatite (green) projected on horizontal plane. Black lines – boreholes. The map was generated by Micromine 2016, commercial license (http://www.micromine.com).

The automatically built maps do not show vein complexes; however, some of them can play a significant role in understanding the deposit structure. So, we showed a zone of dolomite carbonatites manually. This zone was delineated based on our more than twenty-years’ observations^15^, and the borehole data collected from 2008 to 2012. The borehole intervals with dolomite carbonatite projected on horizontal plane are shown in Fig. 7. Direction of linear dolomite veins conforms to the regional North-East fault intersecting the Kovdor massif. The north-eastern end of the fault controls the alkaline-ultrabasic massif Maliy Kovdor. Please note the general concordance of the central oval structure consisting of calcite-rich phoscorites with the dolomite carbonatite zone (see Fig. 6).

A testing set consisted of 276 samples (drillcore intervals and corresponding thin sections), which were excluded from ‘learning’ of the classification system because of their inhomogeneity. Quality of prediction was defined as percent of coincidences of mineral indexes (C, M, A, F) of rock type between predicted from chemical composition and manually measured by thin sections. The coincidences are shown in Table 3.

**Table 3:** Quality of prediction of mineral indexes by bulk-rock chemistry, testing set. Percent of coincindence.

	C	M	A	F
Mineral norms	51	56	61	46
Multiple regression	44	51	49	44
Neural network	66	61	62	61
Logical evaluation	68	61	61	55

## Discussion

### Advantages and limitations of the approach

**Figure 8:**
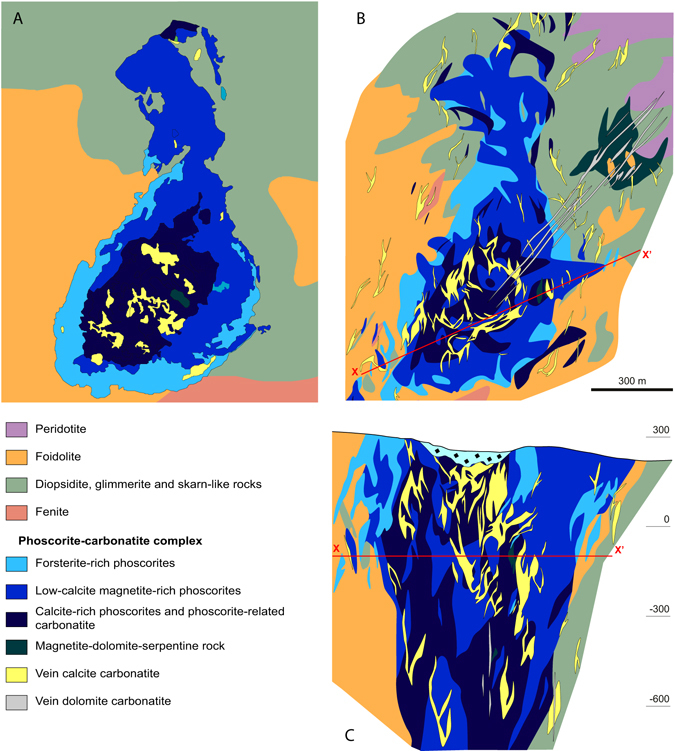
Manually created maps and cross-section of the Kovdor magnetite-apatite-baddeleyite deposit: (**A**) by geological service of JSC “Kovdorskiy GOK”^15^, level ≈−60 m; (**B**,**C**) by P.M. Goryainov and colleagues^13^, level −100 m.

In Figs 6 and 8, we compared the automatic maps with manually built maps^13, 15^. One can see a good general similarity between maps created by neural network classification (Fig. 6C), by logical evaluation of rock types (Fig. 6D), on one hand, and the manual maps published in refs 13 and 15 (Fig. 8), on other hand. The map shown in (Fig. 8A) is based on routine documentation of the Kovdor “Zhelezny” (Iron) quarry, and exploration borehole data; it reflects actual data as of January 1, 2002. The map shown in (Fig. 8B) is based on the borehole data collected during 1940–2011, and our more than twenty-years’ observations over the quarry operation. So, these two maps are considered to be the most recent and representative. The most evident distinction between the automatic map and the recent “manual” maps is the absence of carbonatite vein stockwork. In fact, the method does not generate any geological bodies smaller than the average sampling interval (16 m in the Kovdor deposit case). Average distance between borehole profiles seems to be a “resolution threshold” for linear object (i.e. faults); in our case, it is 50 m. So, there is no abundant carbonatite stockwork pattern except for the largest bodies, and no complicated fault network is presented on N.I. Krasnova’s map^16, 17^.

We examined the modelling procedure to find a potential error source. The primary error source (excluding, certainly, sampling and analytical problems) is classification of rock types by their chemical composition. It appears that we cannot use the method if there are no precise classification boundaries between different types of rocks. But this problem can result from inadequate petrologic classification, i.e. absence of the precise classification boundaries can be a reason for petrologic classification revision. In case of the Kovdor deposit, only 9 of 274 samples were outliers.

One can see spherical “bodies” on peripheries of horizontal and vertical sections (see Figs 5 and 6). Obviously, they appear due to sampling heterogeneity resulting in interpolation errors, i.e. if the distance between samples was more than 150 m (search radius), a sphere with radius 150 m was generated. It means that errors in the automatic 3D geological model can be mainly caused by interpolation inaccuracy. So, the method application requires a sufficiently close and homogenous sampling network, as well as accurate geostatistical studies ensuring reduction of unavoidable sampling incompleteness.

The second possible source of errors is underestimation of faults. Usually information about faults location is available from historical data. If not, interpolation pattern of rock bulk-chemistry can provide this information: very close convergence of interpolation isolines can indicate faults rather accurately. Comparison with other geological maps created by different geologists shows that there are no problems with fault underestimation in the Kovdor deposit case (Figs 6 and 8).

A comparison of prediction quality over the test sample (Table 3) shows that the norms (mineral normative composition) calculation well predicts only apatite content and, to a lesser degree, magnetite content. This is due to the fact that Fe_magn_ and P_2_O_5_ are associated only with magnetite and apatite, respectively. Norms calculation works bad for forsterite because, besides forsterite, there are other silicates in the deposits – phlogopite, diopside, aegirine, nepheline etc. Causes of bad prediction of calcite are more complex. A main cause, we believe, is that the phoscorite-carbonatie complex and surroundings are cut by numerous thin carbonatite veinlets, and probability of presence of the veinlet in a drillcore interval is higher than in thin-section. A second probable cause is that besides calcite there are dolomite and other carbonates (magnesite, siderite, ancylite, etc.) in the complex, which have other ideal CO_2_ content.

Prediction by a multiple regression is worst. We suppose that it is because of the closure problem of chemical and mineral composition of rock (i.e. the sum of components is 100), and linear statistical methods do not work properly, although we have used centered logratio transformation of bulk-chemistry. Probably, this type of transformation is not suited for multiple regression because the centered logratio transformation forms singular numerical system, i.e. sum of all variables, as well as determinants of covariance and correlation matrices are necessarily zero^24^.

Predictions by artificial neural network and by logical evaluation are the best (Table 3 and Fig. 6). Quality of prediction of magnetite and apatite contents are nearly equal. Logical evaluation is better for the calcite prediction, while artificial neural network – for the forsterite prediction.

In general, four used techniques belong to three prediction approaches. Mineral norm calculations are based on *a priori* model. Multiple regression is a linear statistical method. Logical evaluation of rock type is a search of optimal border of rock types (we go through the samples one by one and shift the delineations until they fit at best), i.e. it is a sort of supervised learning or ‘pattern recognition’ (besides mentioned methods, there are random forests, support vector machines, kernel estimators, decision tree learning, maximum entropy classifier and others methods in the field of supervised learning^25, 26^). Artificial neural network and logical evaluation of rock type are supervised classification approaches well established in the field of machine learning sciences. The comparison of prediction quality (Table 3 and Fig. 6) suggests that supervised learning approach is the best for chemistry-to-mineral conversion and automatic 3D mapping.

### Applications of the approach

A real spatial structure is required for comparison of the geological body form with modeled structure of explosion pipes, magmatic eruptive columns, percolation media etc. Estimation of the real and model forms proximity produces constraints for genesis reconstruction. Automatic geological modelling of the Kovdor deposit enables to avoid any impacts resulted from *a priori* genetic conception and subjective factors. The model is based on actual data, consequently, it will be close to real spatial structure of the phoscorite pipe. It allows us to compare qualitatively and quantitatively natural and experimental objects. For example, one can see an analogy between a vertical section of the deposit (Fig. 6C,D, bottom) and a typical structure of rapidly decompressed glasses and rocks: there is a “funnel” formed by calcite-rich phoscorites and surrounded by low-calcite phoscorites. It is similar to the structures developing on the glass decompression front^27^ and bowl-shaped structures in shock decompressed rhyolite^28^. Location of carbonatite body (i.e. usually porous body with minimal density, from −400 m to −800 m on the vertical sections) is similar to the location of bubbles at the depth of 8 cm in shock decompressed glasses^27^. Besides, diagonal orientation of the carbonatite body resembles fractures (i.e. volumes with minimum density) in rapid decompressed pumice^29^. General subvertical dip of ore bodies and concentric differentiation of south part of the deposit is similar to rapidly decompressed, partly crystallized ore melt^30, 31^. Vortex-like structures are also typical for decompressed solid-gas columns, e.g. ref. 32. The series of analogies can be expanded. In future, we plan to perform detailed qualitative and quantitative comparison of the phoscorites bodies’ forms and model objects.

Another application of the proposed approach is geometallulgical modelling. We have obtained 17 types of rocks, 12 of which contain considerable quantities of major ore minerals (apatite and magnetite) in different proportions: CMA, CM, CA, CMF, CAF, CMAF, Magnetitite, MAF, MA, Apatitite, MF and AF. In these rock types variations of ore minerals content can be too high to enable accurate feed ore quality control at the plant. For example, the content of magnetite and apatite in the MA ore (Fig. 3) may vary within 7–95 modal %; herewith the content of the main non-ore minerals here (calcite and forsterite) is less than 10 modal %. However, if necessary, these variations can be significantly reduced. For example, the MA ore can be divided into the necessary number of sub-types by recalculation of P_2_O_5_ into apatite, and Fe_magn_ into magnetite. The same detalization procedure can be easily used for almost all other types of ore, including carbonatites (which can be divided into “ferrous” and “phosphorous” sub-types). Some difficulties may appear only during detalization of the CMAF ore. Therefore, we can conclude that the produced 3D geological model of the Kovdor deposit is a preliminary geometallurgical model that can be developed in more details based on available data as well as comparison of processing products with *in situ* ore. The detailed geometallurgical modelling is in our further plans.

## Conclusions

1. The approach of automatic 3D geologic modelling was introduced. The approach enables to use borehole core bulk-analyses only, without any *a priori* genetic concept or geologic rules (e.g. age sequence). The approach requires a small number of variables (4 in the Kovdor deposit case), but a rather close and homogenous sampling network. Rocks of phoscorites–carbonatites series are well distinguished by their chemical composition according to the classification tetrahedron^14^.
2. The best prediction technique in the framework of the suggested approach is supervised learning. Calculation of normative mineral composition (norms) and linear statistical methods (multiple regression) are likely unsuitable.
3. Automatically built 3D geological models (built by rock type prediction by neural network and logical evaluation) demonstrate good convergence with the most recent and representative maps: the map created by geological service of JSC “Kovdorskiy GOK”^15^ and the map based on borehole data^13^.
4. The approach does not allow generating vein stockwork patterns or other geological details smaller than the average length of a sampling interval (16 m in the Kovdor deposit case).
5. The proposed approach can be a tool of preliminary geometallulgical modelling.

## Materials and Methods

The studies were based on the results of rocks chemical analyses implemented during exploration of deep horizons at the Kovdor deposit (in 2008–2012). The total length of analyzed core samples is 30213 m taken from 108 exploratory boreholes, number of sample intervals is 1846, and average length of the intervals is about 16 m. Analyses for SiO_2_, TiO_2_, MgO, CaO, K_2_O, Na_2_O, Al_2_O_3_, FeO_total_, Fe_magn_, P_2_O_5_, ZrO_2_, S_total_, and CO_2_ were carried out. Analyses were performed in analytical laboratory of JSC “KGILC” (Apatity, Murmansk region, Russia) with a conventional wet chemistry method (different types of titration and gravimetric analyses). Fe_magn_ means metallic iron content in mineral fraction separable by magnetic separation.

Percentage of rock-forming minerals (carbonates, magnetite, apatite, forsterite) was calculated for 550 samples representing all varieties of phoscorites, carbonatites and main types of host rocks, with optical microscope in thin sections. Precise diagnostics of minerals was carried out using LEO-1450 scanning electron microscope featuring the Röntek energy-dispersive microanalyzer. Statistical analysis was carried out with the program STATIATICA 8 (StatSoft), geostatistical investigations – Micromine 16, logical computations – the Gnumeric Spreadsheet 1.10.16 (The GNOME Project, http://www.gnumeric.org).

To avoid the closure problem associated with this kind of data (the chemical variables are not independent because they are confined within a closed composition), we used centered log-ratio transformation^23^ of the data for statistical analyses (multiple regression and artificial neural network).

Interpolation was performed by ordinary kriging. For weight equalization, drillcore interval samples were preliminarily composited on the basis of the Fe_magn_ grade they contain; minimal length is 5 m and maximal is 10 m. Data were interpolated in the empty block model created by JSC “RJC” (St. Petersburg, Russia) for Kovdor deposit. Its characteristics are the following: block size — 15 × 15 × 15 m; Easting (Y) minimum is −4000 m, maximum is −1000 m; Northing (X) minimum is −2000 m, maximum is 1000 m; Elevation (Z) minimum −1605.5 m, maximum is −347.5 m; totally 4703979 blocks. After the variables were interpolated into the basic block model, empty blocks were deleted.

The main axis of kriging ellipsoid is vertical in all interpolation models, the search figure is spherical; the sphere is divided into 4 sectors; maximal points per sector is 5; negative kriging weights are nulled. To avoid a problem of kriging smoothing and edge oscillation, interpolation was performed in three runs with an increasing search radius (25, 50 and 150 m) and decreasing minimal interpolation points (4, 3 and 1, respectively). Distributions of Fe_magn_ and P_2_O_5_ are normal, and CO_2_ and SiO_2_ are lognormal, so we use natural logarithms of CO_2_ and SiO_2_ contents for kriging with back transformation after interpolation. Interpolation parameters of used components are listed in Table 4.

**Table 4:** Parameters of bulk-rock chemistry interpolation by ordinary kriging of the Kovdor phoscorite-carbonatite complex.

Component	Distribution of raw data	Additive constant for lognormal transformation*	Nugget-effect	Range, m	Sill	Semivariogram type
Fe_magnetic_	Normal	No transformation	6.9	131	91.1	Spherical
SiO_2_	Lognormal	0	0	150	0.436	Spherical
P_2_O_5_	Normal	No transformation	0	111	10.55	Exponential
CO_2_	Lognormal	0	0.079	139	0.515	Spherical

^*^In case of lognormal distribution of raw data, i.e. SiO_2_ and CO_2_.

## Electronic supplementary material

Supplementary Dataset 1

Supplementary Dataset 2
